# IN.PACT Amphirion paclitaxel eluting balloon versus standard percutaneous transluminal angioplasty for infrapopliteal revascularization of critical limb ischemia: rationale and protocol for an ongoing randomized controlled trial

**DOI:** 10.1186/1745-6215-15-63

**Published:** 2014-02-19

**Authors:** Thomas Zeller, Iris Baumgartner, Dierk Scheinert, Marianne Brodmann, Marc Bosiers, Antonio Micari, Patrick Peeters, Frank Vermassen, Mario Landini

**Affiliations:** 1 Department of Angiology, Universitäts Herzzentrum Freiburg Bad Krozingen, Bad Krozingen, Germany; 2 Swiss Cardiovascular Center, Division of Angiology, University Hospital, Inselspital, Bern, Switzerland; 3 Center of Vascular Medicine, Park Hospital Leipzig, Leipzig, Germany; 4 Department of Angiology, Medical University Graz, Graz, Austria; 5 Department of Vascular Surgery, A.Z. Sint-Blasius, Dendermonde, Belgium; 6 Invasive Cardioangiology GVM Care and Research, Palermo, Italy; 7 Department of Cardiovascular & Thoracic Surgery, Imelda Hospital, Bonheiden, Belgium; 8 Department of Vascular Surgery, Ghent University Hospital, Ghent, Belgium; 9 Department of Medical Affairs, Endovascular Therapies, Medtronic Cardiovascular, Santa Rosa CA, USA

**Keywords:** Peripheral vascular disease, Critical limb ischemia, Infrapopliteal, Drug-eluting balloon

## Abstract

**Background:**

The effectiveness and durability of endovascular revascularization therapies for chronic critical limb ischemia (CLI) are challenged by the extensive burden of infrapopliteal arterial disease and lesion-related characteristics (e.g., severe calcification, chronic total occlusions), which frequently result in poor clinical outcomes. While infrapopliteal vessel patency directly affects pain relief and wound healing, sustained patency and extravascular care both contribute to the ultimate “patient-centric” outcomes of functional limb preservation, mobility and quality of life (QoL).

**Methods/Design:**

IN.PACT DEEP is a 2:1 randomized controlled trial designed to assess the efficacy and safety of infrapopliteal arterial revascularization between the IN.PACT Amphirion™ paclitaxel drug-eluting balloon (IA-DEB) and standard balloon angioplasty (PTA) in patients with Rutherford Class 4-5-6 CLI.

**Discussion:**

This multicenter trial has enrolled 358 patients at 13 European centers with independent angiographic core lab adjudication of the primary efficacy endpoint of target lesion late luminal loss (LLL) and clinically driven target lesion revascularization (TLR) in major amputation-free surviving patients through 12-months. An independent wound core lab will evaluate all ischemic wounds to assess the extent of healing and time to healing at 1, 6, and 12 months. A QoL questionnaire including a pain scale will assess changes from baseline scores through 12 months. A Clinical Events Committee and Data Safety Monitoring Board will adjudicate the composite primary safety endpoints of all-cause death, major amputation, and clinically driven TLR at 6 months and other trial endpoints and supervise patient safety throughout the study. All patients will be followed for 5 years. A literature review is presented of the current status of endovascular treatment of CLI with drug-eluting balloon and standard PTA. The rationale and design of the IN.PACT DEEP Trial are discussed. IN.PACT DEEP is a milestone, prospective, randomized, robust, independent core lab-adjudicated CLI trial that will evaluate the role of a new infrapopliteal revascularization technology, the IA-DEB, compared to PTA. It will assess the overall impact on infrapopliteal artery patency, limb salvage, wound healing, pain control, QoL, and patient mobility. The 1-year results of the adjudicated co-primary and secondary endpoints will be available in 2014.

**Trial registration:**

NCT00941733

## Background

Percutaneous transluminal angioplasty (PTA) is an established alternative to open surgical bypass for the treatment of infrainginual disease of critical limb ischemia (CLI) patients with limited life expectancy, multiple surgical comorbidities, and/or those who lack an adequate venous conduit [1-3]. Several studies established lower peri-procedural mortality and morbidity and high technical success for PTA, leading some centers to consider infrapopliteal artery angioplasty, particularly in specific high-risk CLI patient cohorts, to be the “first line” therapeutic approach [4-7]. Conversely, the extent and burden of infrapopliteal arterial disease, particularly in diabetics, are well described [8], and high post-PTA restenosis rates have been consistently reported [9-11]. Nonetheless, reports that include adjudication of restenosis rates by an independent angiographic core lab as part of a large multicenter randomized controlled trial are lacking. Likewise, the impact of high infrapopliteal vessel restenosis rates on the prognosis of CLI patients is unclear. Yet, despite concerns of high infrapopliteal PTA restenosis rates, the limb salvage rates appear acceptable [10] when compared to open lower limb bypass [12]. Thus, there is an evolving impression, endorsed by societal guidelines [13,14], that the potential near-term benefit of the less invasive PTA approach in CLI patients, when compared to surgery, favors PTA as the “first line approach” in specific patients. The potential negative impact on limb salvage of a required percutaneous reintervention or surgical bypass after a failed PTA remains, however, a concern. Recent data suggest that patients who experienced tibial PTA restenosis and who subsequently underwent a lower limb bypass required more distal target anastomoses [15] and had higher 1-year amputation and graft closure rates [16] when compared to patients without prior PTA. Likewise, the BASIL trial investigators observed that patients undergoing bypass after a failed PTA had worse amputation-free survival (AFS) rates than those undergoing primary bypass [3]. Therefore, there is an evolving concern that CLI patients followed for a longer time period will demonstrate high tibial PTA restenosis rates that may ultimately require lower extremity bypass, which are, in turn, associated with higher bypass failure rates and worse outcomes [16].

Paclitaxel drug-eluting balloons are designed to promote arterial patency by reducing neointimal proliferation. In prospective clinical trials in relatively small CLI patient cohorts, tibial vessel drug-eluting balloon (DEB) angioplasty was associated with significantly reduced restenosis rates and late lumen loss (LLL) at 3, 6, and 12 months [17-19]. Although these angiographic assessments were not core lab adjudicated, there is a growing perception that the clinical results of DEB tibial angioplasty mirror the clinical experience in the superficial femoral artery in claudicants [20-23]. However, the potential for tibial artery DEB angioplasty to provide a more durable patency rate and, thereby, potentially favorably impact limb salvage, wound healing, time to wound healing, pain control, quality of life (QoL) indices, and mobility has yet to be defined in a prospective, robust, randomized assessment compared to PTA. IN.PACT DEEP was initiated as the first trial to systemically assess the Safety and Efficacy of the new IN.PACT Amphirion™ paclitaxel drug-eluting balloon (IA-DEB) technology in CLI patients with infrapopliteal disease.

### Hypothesis

We hypothesize that the IA-DEB will significantly reduce angiographically assessed target lesion late lumen loss (LLL) compared to standard PTA in infrapopliteal lesions up to 10 cm in length and reduce clinically driven target lesion revascularization (TLR) in amputation-free surviving CLI patients through 12 months. Moreover, we assumed that the IA-DEB would be non-inferior to PTA with reference to the rate of all-cause death, major amputation, and clinically driven TLR through 6 months.

## Methods/Design

### Trial design

**Figure 1:**
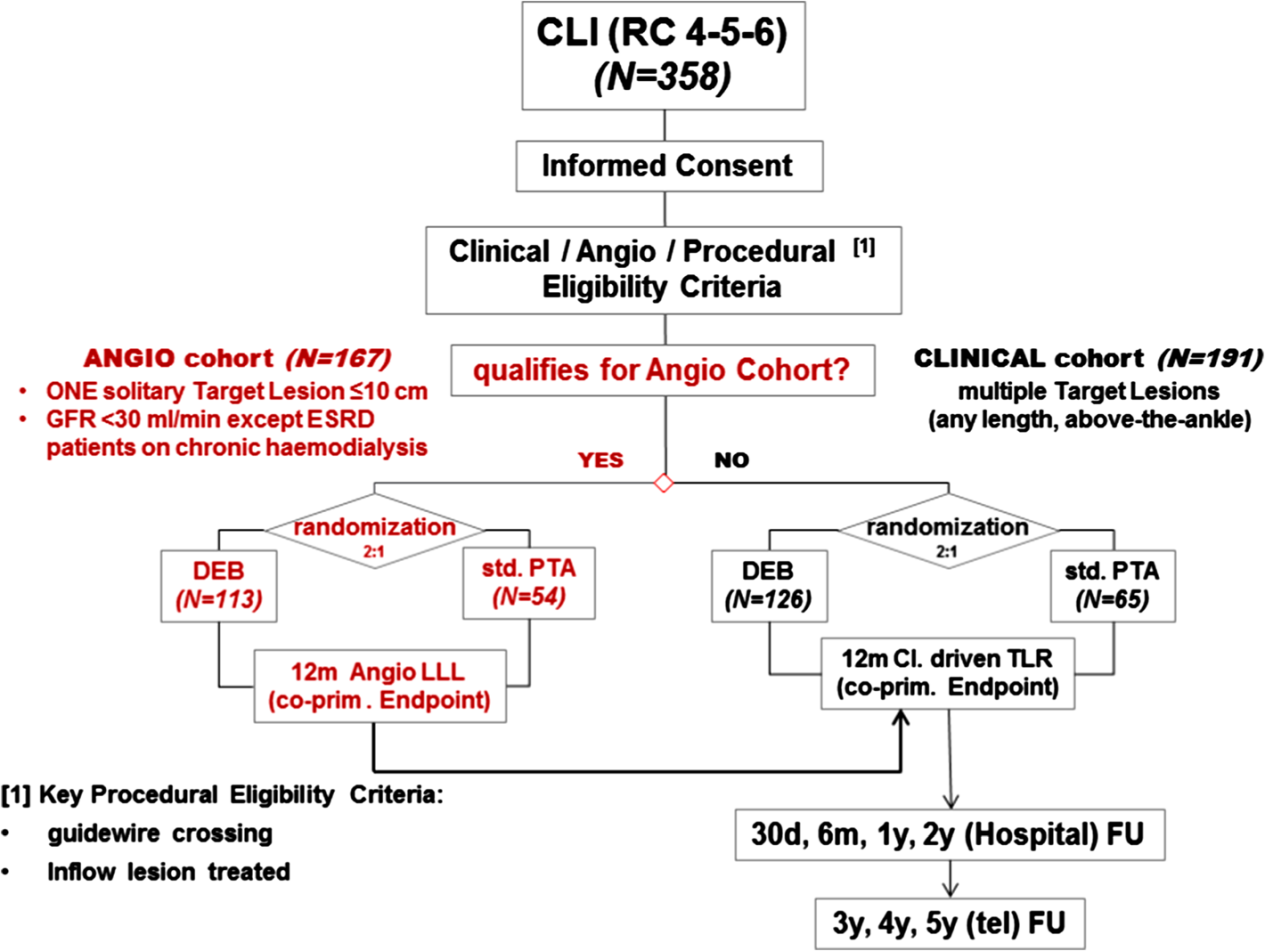
IN.PACT DEEP treatment cohort assignment/randomization flowchart.

This trial is a 2:1 randomized, controlled, patient-blinded multicenter trial that compares the IA-DEB to PTA in terms of angiographically assessed target lesion LLL and clinically driven TLR of infrapopliteal arteries in Rutherford class 4-5-6 symptomatic critical limb ischemia patients through 12 months (Figure 1). Consecutive patients matching all General Eligibility Criteria (Table 1 Sections A-D) were consented for the trial. All patients were required to be free of further general procedural exclusion criteria (Table 1 Section E) such as unsuccessful crossing of the target lesion with a guide wire.

**Table 1:** IN.PACT DEEP inclusion and exclusion criteria

	
(A) General inclusion criteria	
	i.1 Age ≥ 18 years and ≤85 years
	i.2 Patient or patient’s legal representative has been informed of the nature of the study, agrees to participate, and has signed an EC-approved consent form
	i.3 Female patients of childbearing potential have a negative pregnancy test ≤7 days before the procedure and are willing to use a reliable method of birth control for the duration of study participation
	i.4 Patient has documented chronic critical limb ischemia (CLI) in the target limb prior to the study procedure with Rutherford category 4, 5, or 6
	i.5 Life expectancy >1 year in the investigator’s opinion
(B) General exclusion criteria	
	e.1 Patient unwilling or unlikely to comply with follow-up schedule
	e.2 Planned major index limb amputation
(C) General angiographic inclusion criteria	
	i.6 Reference vessel diameter(s) between 2 and 4 mm
	i.7 Single or multiple lesions with ≥70% DS of different lengths in one or more main afferent crural vessels including tibioperoneal trunk
	i.8 At least one non-occluded crural vessel with angiographically documented run-off to the foot either directly or through collaterals
(D) General angiographic exclusion criteria	
	e.3 Lesion and/or occlusions located in or extending to the popliteal artery or below the ankle joint space
	e.4 Inflow lesion or occlusion in the ipsilateral iliac, SFA, or popliteal arteries with length ≥15 cm
	e.5 Significant (≥50% DS) inflow lesion or occlusion in the ipsilateral iliac, SFA, or popliteal arteries left untreated
	e.6 Previously implanted stent in the TL(s)
	e.7 Aneurysm in the target vessel
	e.8 Acute thrombus in the TL
(E) General procedural exclusion criteria	
	e.9 Failure to obtain <30% residual stenosis in pre-existing, hemodynamically significant (≥50% DS and <15 cm length) inflow lesions in the ipsilateral iliac, SFA, or popliteal artery. DES and/or DEB was not allowed for the treatment of inflow lesions
	e.10 Failure to cross the TL with a 0.014′ guide wire
	e.11 Use of alternative therapy, e.g., atherectomy, cutting balloon, laser, radiation therapy, DES as part of the index procedure
(F) Angiographic cohort angiographic inclusion criteria	
	a.i.1 Angio-TL is one identifiable single solitary or a series of multiple adjacent lesions with a DS ≥ 70% and a cumulative length ≤ 100 mm that can be covered by a single IN.PACT Amphiron™ (10-mm balloon landing zone in both edges is mandatory)
	a.i.2 Angio-TL is the only lesion in that vessel (only 1 Angio-TL per patient is allowed)
(G) Angiographic cohort general exclusion criteria	
	a.e.1 GFR <30 ml/min except for patients with renal end-stage disease on chronic hemodialysis

This trial is composed of two subject cohorts (Clinical cohort and Angio cohort), the second characterized by more restrictive eligibility criteria than the first: subjects who met certain additional and specific eligibility criteria and avoided specific exclusion criteria (Table 1 Sections F and G) were allocated to an “Angio” cohort; the others were allocated to the “Clinical” cohort. Finally, subjects were randomized 2:1 to IA-DEB or PTA within their assigned cohort. All subjects from both cohorts will be part of the clinical assessment and followed for 5 years post-randomization. Only subjects from the Angio cohort will undergo angiographic evaluation at 12 months. In August 2012, the IN.PACT DEEP Trial completed enrollment of 358 subjects (Figure 1).

### Primary efficacy endpoints

The IN.PACT DEEP trial was designed before there was preliminary evidence of the degree of efficacy of DEB relative to PTA in the infrapopliteal vasculature. It was therefore decided to choose a method capable of distinguishing even subtle differences in performance. LLL assessment is considered the most sensitive, objective determinant of post-intervention vessel narrowing; this methodology was accordingly selected as the best arbiter of a potential DEB treatment effect. Thus, an angiographic core lab-adjudicated infrapopliteal artery target lesion LLL at 12 months post-intervention (applying to the Angio cohort only) and clinically driven TLR through 12 months (applying to the entire subject population) were selected as co-primary endpoints to provide assessment of vessel patency in the two treatment cohorts from both angiographic and clinical perspectives. “Clinically driven” TLR is defined as (1) directed by an increase in size of a pre-existing wound(s) and/or (2) occurrence of a new wound(s), and/or (3) deterioration in Rutherford class. To assist in quantitation of wound healing assessments, all investigational sites were provided with and trained on the use an electronic wound reader able to objectively record the morphological status of wound’s area and depth (SilhouetteMobile™, Aranz Medical Ltd., Auckland, New Zealand). An independent Wound Core Lab. (SynvaCor, Springfield, IL) subsequently adjudicated data. USA. Important “patient-centric” secondary endpoints to be assessed include time to wound healing, change in ischemic pain, quality of life (QoL), and walking capacity. Other secondary endpoints include amputation-free survival (AFS), major adverse events (MAE) and sustained clinical improvement. The primary safety endpoint is described below; secondary endpoints and major inclusion/exclusion clinical and angiographic criteria are detailed in Tables 1 and 2, respectively.

**Table 2:** IN.PACT DEEP trial secondary endpoints

	
(1)	Amputation-free survival at 30 days, 3 and 6 months, 1, 2, 3, 4 and 5 years
(2)	Rate of wound healing at 30 days, 6 months, 1 and 2 years
(3)	Amputation-free survival and wound healing at 6 months, 1 and 2 years
(4)	Amputation-free survival and resolved CLI at 6 months, 1 and 2 years
(5)	Death, amputation, and clinically driven TLR at 30 days, 6 months, 1 and 2 years
(6)	Primary sustained clinical improvement: an improvement shift in the Rutherford classification of 1 class in amputation-free, clinically driven TLR-free surviving patients at 1 year
(7)	Secondary sustained clinical improvement: an improvement shift in the Rutherford classification of 1 class including the need for clinically driven TLR in amputation-free surviving patients at 1 year
(8)	QoL assessment by EQ5D at 6 months, 1 and 2 years vs. baseline
(9)	Walking capacity assessment by WIQ at 6 months, 1 and 2 years
	MAE at 30 days, 6 months, 1, 2, 3, 4, and 5 years
(10)	Device success defined as the exact deployment of the device according to the instructions for use as documented with suitable imaging modalities and, in the case of digital subtraction angiography, in at least two different imaging projections
(11)	Technical success defined as successful vascular access and completion of the endovascular procedure and immediate morphological success with ≤50% residual diameter reduction of the treated lesion on completion angiography
(12)	Procedural success defined as combination of technical success, device success and absence of procedural complications
(13)	For the Angio cohort: improvement in 12 months of percent diameter stenosis (%DS) of the TL assessed by quantitative vascular angiography
(14)	Days of hospitalization

### Subjects

A total of 358 patients with symptomatic Rutherford class 4-5-6 CLI who matched the trial eligibility criteria were randomized 2:1 to treatment with IA-DEB (*N* = 239) or PTA (*N* = 119). One hundred sixty-seven of these subjects were allocated to the Angio cohort (113 subsequently randomized to IA-DEB and 54 to PTA) and the remaining 191 to the Clinical cohort (126 subsequently randomized to IA-DEB and 65 to PTA). LLL angiography will be assessed at the 12-month follow-up for the Angio cohort only and clinically driven TLR through 12 months will be assessed in all subjects. All subjects will be evaluated yearly for 5 years.

### Eligibility criteria

Subjects were required to meet all general, angiographic and procedural eligibility criteria (Table 1A-E; see below) to be considered for the trial. If any of the exclusion criteria (Table 1B and E) were met, the subject was excluded from the trial.

Subjects who met certain specific additional inclusion and exclusion criteria (see Figure 1 and Table 1F and G) were allocated to the Angio cohort. A maximum lesion length of 10 cm was selected for this cohort to facilitate the technical assessment of LLL by the blinded core lab.

### Extent of infrapopliteal revascularization and wound healing

The angiosome concept, introduced by Taylor et al. [24], is an anatomic unit of tissue, fascia, muscle, and bone fed by a source artery and drained by specific veins. The lower extremity and foot are divided into six specific angiosomes fed by the posterior tibial artery (3 angiosomes), anterior tibial artery (1 angiosome), and peroneal artery (2 angiosomes). Alterations in regional pedal circulation impact successful healing in patients with ischemic ulcers [25,26]. Regional perfusion differences may be accentuated if the source or wound-related artery (WRA) to the ischemic angiosome is occluded and collateral vessels perfuse the impacted angiosome indirectly.

Various contributing factors that influence successful revascularization and impact wound healing have been described. Iida [25] and Neville [27] demonstrated significantly improved wound healing and rates of freedom from amputation when direct pulsatile flow via the WRA was established compared to when indirect revascularization was implemented. Peregrin [28] observed that better outcomes were achieved with revascularization of a greater number of vessels, while Faglia [29] reported that perfusion via the anterior and/or posterior tibial arteries generally resulted in better outcomes when compared to peroneal artery recanalization alone. Still better outcomes were achieved with revascularization of a greater number of vessels. Additionally, the importance of foot vascular anatomy [30], pedal circulation [31], and micro-circulation [32] to CLI outcomes has been recently emphasized. Ultimately, the revascularization target and extent of the infrapopliteal/pedal revascularization remain an area of great interest and debate in CLI treatment. To address these issues, the IN.PACT DEEP trial will evaluate the impact of direct vs. indirect revascularization and the number of revascularized vessels on wound healing and limb salvage. This prospective evaluation consented subjects prior to the confirmation of full eligibility, which included some angiographic and procedural criteria. Subjects who failed angiographic and procedural eligibility criteria were considered “screen failures”. The screen failures were not considered as enrolled subjects for the purposes of intent-to-treat (ITT) analysis.

### Randomization

Randomization of subjects proceeded after all procedural and angiographic eligibility criteria had been met, including the requirements that all inflow lesions had been successfully treated and that the guidewire had successfully crossed the target lesion and was positioned into the distal true lumen (Table 1). The randomization process was performed using blocks of sealed envelopes.

### Ethics

This trial is conducted in accordance with the principles of the Declaration of Helsinki, ISO 14155 and Good Clinical Practices guidelines. The Ethics Committees of all investigational sites (Table 3) approved the trial protocol, and written informed consent was obtained from all subjects before enrollment. Subjects and their treating physicians retained the right to withdraw from the trial and all follow-ups at any time without prejudice.

**Table 3:** Ethics committees used in the IN.PACT DEEP trial

**Site name**	**Ethics committees**
Park-Krankenhaus Leipzig-Südost GmbH/Herzzentrum Leipzig GmbH	Ethik Kommission, Härtelstrasse 16–18, 04107 Leipzig
University of Bern Angiology Division	Nationale Ethikkommission Bern, Generalsekretärin, Postfach 56, CH-3010 Bern
Medical Care Center Prof. Mathey, Prof. Schofer GmbH	FEKI (Prof H.P. Graf MD, PhD) Nationale Ethikkommission, Mozartstrasse 21, DE-79104 Freiburg
University of Heidelberg	
Herz-Zentrum Bad Krozingen Angiology	
A.Z. Sint-Blasius Vascular Surgery	Universitair Ziekenhuis Gent, Commissie voor Medische Ethiek, De Pintelaan 185B, BE 9000 Ghent
Imelda Hospital Cardiovascular & Thoracic Surgery	
Ghent University Hospital Vascular Surgery	
Zol St-Jan	Commissie Medische Ethiek, Schiepse Bos 6, BE-3600 Genk
Medical University Graz	Ethikkommission, Univ.Prof. DI Dr. Haas, Aenbruggerplatz 2, A-8036 Graz
Villa Maria Eleonora Hospital	Comitato Bioetico, Aziendale, Via G. Cusmano n.24, 90141 Palermo
Luzerner Kantonsspital	Präsident der Ethik Kommission des Kantons Luzern, Luzerner Kantonspital, 6000 Lucerne 16
St. Antonius Hospital	VCMO ST Antoniusziekenhuis, Koekoekslaan 1, 3435 CM Nieuwegein

### Safety and quality control

#### Data safety monitoring board

A Clinical Events Committee (CEC) will assess the composite primary safety endpoints of all-cause death, major amputation, and clinically driven TLR through 12 months and will adjudicate other trial endpoints. A Data Safety Monitoring Board (DSMB) will periodically review safety data for subject safety, the study conduct, and progress.

#### Adverse and serious adverse events

**Figure 2:**
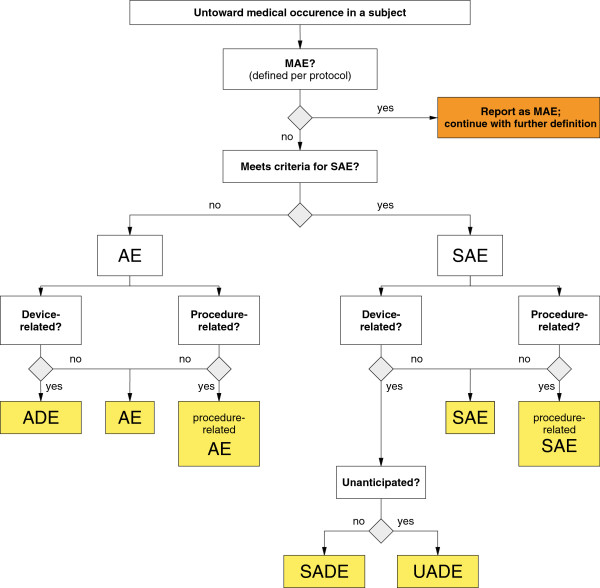
Adverse events categorization flowchart.

Adverse events (AEs) are defined as any untoward medical occurrence in a subject whether or not considered related to the study device that is identified or worsens during the trial. AEs are classified following the flowchart below (Figure 2).

AEs will be assessed and documented by the site investigators at the time of the procedure and at all follow-up visits (scheduled and unscheduled). All suspected AEs will be recorded on the AE Log in the electronic clinical report form (e-CRF). An adverse device effect (ADE) is any untoward and unintended response to a medical device including any event resulting from insufficiencies or inadequacies in the instructions for use or the deployment of the device as well as any event that is a result of a user error.

AEs will be classified on the AE Log of the e-CRF as follows, with categories 3 to 5 considered to meet the definition of a serious adverse event (SAE): (1) no therapy, no consequence; (2) nominal therapy, no consequence; (3) required intervention to prevent serious outcome; (4) hospitalization or prolongation of hospitalization; (5) persistent/significant disability/incapacity life-threatening/death. Major or minor wound debridement is not considered an AE. SAEs include AEs of categories 3–5 and include any untoward medical occurrence in a subject which:

1. Led to a death or

2. led to a serious deterioration in the health of the subject that:

a. resulted in a life-threatening illness or injury, and/or

b. resulted in a permanent impairment of a body structure or body function, and/or

c. required subject hospitalization or prolongation of existing hospitalization and/or

d. resulted in a medical or surgical intervention to prevent permanent impairment to body structure or body function.

A serious adverse device effect (SADE) is an adverse effect that results in any of the consequences characteristic of an SAE or that might have led to any of these consequences had suitable action not been taken, had intervention not been made, or had circumstances been less opportune.

An unanticipated adverse device effect (UADE) is defined as (1) any serious adverse effect on health or safety or any life-threatening problem or death caused by or associated with the use of the investigational device if that effect, problem, or death was not previously identified in nature, severity, or degree of incidence in the investigational plan or application (including a supplementary plan or application) or (2) as any other unanticipated serious problem associated with a device that relates to the rights, safety, or welfare of subjects.

In addition to the “generic” AE Log, dedicated sections are present in the e-CRF allowing tracking of specific events as follows: (1) all TLR; (2) all target extremity revascularization (TER); (3) all deaths; (4) all amputations; (5) all UADEs.

MAEs as in Figure 2 are defined as composite rates of all-cause death, minor amputation, and major amputation.

### Statistical analysis

The total sample size for the IN.PACT DEEP trial was calculated at 357 subjects, which fully powers the primary co-efficacy endpoint of LLL (80%) and the primary composite safety endpoint (80%) based on initial estimates of event rates and effect sizes of the two arms, IA-DEB and PTA, randomized 2:1. The primary co-efficacy endpoint of 12-month TLR is powered to 65% with the indicated sample size.

### Primary co-efficacy endpoints

The first primary efficacy endpoint is LLL, assessed at 12 months or at the time of TLR; the second is incidence of clinically driven TLR assessed through 12 months. Each will be tested for superiority in a comparison of the randomized groups.

### Sample size consideration

#### Primary efficacy endpoint: angiographic cohort

The statistical hypothesis of superiority is assessed using a two-sample *t*-test of IA-DEB versus PTA, with 5% two-sided alpha and 80% desired power. Estimates relevant to the LLL primary efficacy endpoint depend on two observations:

1. The hypothesized treatment effect – that is, the mean difference between the IA-DEB and PTA arms at 12 months.

2. An estimate of variability, specifically the standard deviation (SD).

To obtain estimates of these numbers, a meta-analysis of PTA by Romiti et al. [12] was examined for results on (binary) primary patency. The indicated value was 58.1% at 12 months, meaning that 58.1% of enrolled subjects in the studies summarized had binary primary patency (restenosis less than 50%) at 12 months. The value of 58.1% for binary patency was used in a simulation to estimate the mean LLL indexed to reference vessel diameter as follows.

1. The standard deviation of indexed LLL was assumed to be 0.25, which is a reasonably conservative value for a random variable of this type.

2. Indexed LLLs were then simulated as being drawn from a normal distribution such that 58.1% of the cumulative distribution function lay below 0.5, the value that defines binary patency. Based upon the parameters above, the mean for the PTA group is estimated to be 0.45. This is reasonable in that it is somewhat less than 0.5, which would be expected to be the case since more than half of subjects had binary patency.

Assuming a 30% relative reduction in mean indexed LLL in the IA-DEB group compared to the PTA group—that is, a mean in the IA-DEB group of (1–0.3) (0.45) = 0.315—and accounting for 25% potential endpoint attrition (larger than the 10% for safety since death is not included in the endpoint and the period of follow-up is longer), the resulting sample size is 168 (with 112 expected in the IA-DEB group and 56 in the PTA group) subjects in the randomized subgroup qualifying for evaluation of LLL. Note: subjects undergoing bailout or provisional stenting in either randomized arm are considered to continue as normal for endpoint analysis (per ITT principles as below).

#### Primary efficacy endpoint: clinical cohort

For the efficacy endpoint of 12-month TLR, the statistical hypothesis is tested using Fisher’s exact test of proportions of IA-DEB versus PTA, again with 5% two-sided alpha and 80% desired power. For this endpoint, it is assumed that among evaluable subjects (that is, those not lost to follow-up), 50% of PTA subjects will undergo TLR during the 12-month follow-up and that a relative reduction of 30% in TLR will be seen in IA-DEB subjects, resulting in a rate of [(1–0.3) (0.5) = 0.35] 35% for TLR in IA-DEB subjects. After 25% attrition as before, the required sample size is 504.

As this sample size is larger than the intended enrollment for the trial, the power for this endpoint in the current trail based upon intended sample size of 357 is 65% rather than 80%. An interim analysis will be conducted when 150 subjects have attained 12 months of follow-up for the purpose of potentially stopping early for efficacy on this endpoint.

### Primary safety endpoint

The primary safety endpoint for the proposed trial is a composite of all-cause death, major amputation, and clinically driven TLR, tested for non-inferiority using Blackwelder’s [33] method between the randomized groups and evaluated at the 6-month follow-up visit with a non-inferiority margin defined at 10%. For this endpoint, it is assumed that among evaluable subjects (that is, those not lost to follow-up), 40% of PTA subjects will experience an event meeting the safety endpoint by the 6-month follow-up and that a relative reduction of 15% in TLR will be seen in IA-DEB subjects, resulting in a rate of [(1–0.15) (0.4) = 0.34] 34% in IA-DEB subjects. After 10% potential attrition relative to this endpoint, the required sample size is 357 (with 238 expected in the IA-DEB group and 119 in the PTA group). The power for this test is greater than 80%.

### Early trial termination

An interim analysis of 150 subjects at 12 months to assess the primary safety and efficacy endpoints is to be performed by an independent group and maintained confidentially. The objective of this interim analysis is two-fold:

•To permit early stopping of the trial for overwhelming efficacy (and not futility)

•For use in designing and powering a subsequent United Stated Investigational Device Exemption (US IDE) trial.

The interim analysis to assess all of the primary safety and efficacy endpoints will be conducted once 150 subjects (approximately 100 IA-DEB and 50 PTA) have reached their 12-month follow-up visits. This interim look would permit early stopping for efficacy using an O’Brien-Fleming-type alpha-spending function. Based on 150 out of 357 total enrolled subjects in the trial, the two-sided *P*-values required at the interim and final looks will be 0.006 and 0.048, respectively.

Incorporating the Hochberg adjustments, efficacy would be met at the interim look if at least one of the co-primary endpoints is met at a *P*-value of 0.003 or if both are met at 0.006; similarly, at the final look, efficacy will be met if either of the co-primary endpoints is met at a *P*-value of 0.024 or if both are met at 0.048.

### Intervention

Before the initiation of the interventional procedure, a 55-cm radiopaque ruler was affixed to the index extremity extending from tibial tuberosity to the level of the lateral malleolus. This approach ensured adequate angiographic documentation of the tibial lesion treatment zone and assured appropriate angiographic analysis (i.e., LLL) within the treatment zone on all subsequent planned imaging studies and unplanned interventions. Approach to the tibial artery target lesion was contralateral retrograde or ipsilateral antegrade. Accessing the target lesion (TL) from the popliteal or pedal approach was contraindicated. All imaging was to be performed using digital subtraction angiography. If the subject met the pre-specified angiographic cohort eligibility criteria (Table 1), the subject was selectively entered into the angiographic cohort trial arm.

The treatment objective is to obtain a patent infrapopliteal artery target lesion, with uninterrupted blood flow to the foot. Therefore, significant lesions in the aorto-iliac and/or femoropopliteal arterial segments were treated during the index procedure. However, treatment success of the inflow lesion was to be documented as successful (i.e., residual stenosis <30% by visual estimate). This interventional strategy conforms to standard of practice in maximizing inflow into the infrapopliteal segment in CLI patients.

Prior to intervention, digital subtraction angiography of the inflow segment and pedal runoff imaging were required. Standard interventional techniques in crossing arterial stenoses and/or occlusions were to be employed; re-entry devices in the infrapopliteal segment were contraindicated. Pre-dilatation with an undersized (non-drug-eluting) balloon were required in case of total occlusions and sub-occlusive lesions; however, adjuncts to angioplasty, including atherectomy, cutting or scoring balloons were not permitted. The subject was randomized to treatment with the IA-DEB or PTA only after successful traversal of the TL and placement of the guidewire into the distal true lumen. If all requirements were not documented, the subject was considered a screen failure.

After documentation of the above requirements, if the subject was randomized to the investigational treatment, the IA-DEB balloon of a diameter approximating a 1:1 ratio to the reference artery diameter was deployed and inflated for a minimum of 1 min. If deemed necessary by the operator, a post-dilatation of the TL was performed using a standard short-length standard PTA balloon intended to resolve persistent residual stenosis.

Provisional stenting was permitted according to the following specific criteria:

a) Major dissection (grade C or above). However, in order to limit stenting as much as possible, prolonged standard PTA (3 min) with a non-drug-eluting balloon was to be performed first.

b) Occlusive complication (e.g., recoil) that resulted in severely decreased target vessel flow that did not respond to repeated prolonged balloon inflation.

c) Persistent residual stenosis >50% (documented by the worst of 2 orthogonal views after optimal PTA).

In the case of provisional stent implantation, a commercially available bare metal stent could be used. As this is an ITT analysis, subjects receiving provisional stents will be tracked in their respective assigned randomized trial arms. A completion digital subtraction angiogram in two oblique views of the TL and pedal run-off was required.

### Follow-up

Clinical follow-up will proceed as noted in Table 4. All subjects will be followed for 5 years.

**Table 4:** Trial assessment requirements

	**Baseline**	**Procedure**	**Discharge**	**30 days (24-45)**	**3 months (84-105)(84-105)**	**6 months (174-195)**	**1 year (335-395)**	**2 years (700-790)**	**3 years (1065-1125)**	**4 years (1430-1490)**	**5 years (1795-1855)**	**Unsched**
Demography	X											
Medical History												
Physical Exam	X		X	X		X	X	X				X
Concomitant Meds												
Anticoagulant/antiplatelet therapy	X^1^	X^2^	X	X								
Informed Consent	X											
Incl/Excl Evaluation	X	X										
Routine Lab testing (see Table 2)	X^3^		X	X		X	X	X				X
Ankle pressure, toe pressure, TcPO_2_, PVR (at least 1 required) and brachial pressure	X^3^		X	X		X	X	X				X
Wound assessment^7^ and Wound Care	X		X^8^	X		X	X	X				X
Rutherford Staging	X			X		X	X	X				X
EQ5D	X^3^					X	X	X				X
WIQ						X	X	X				X
Angiography	X^3^	X					X^4^					X^5^
Hospital FU Visits				X		X	X	X				
Telephone FU					X				X	X	X	
Adverse Event Assessment		X	X	X	X^6^	X	X	X	X^6^	X^6^	X^6^	X

^1^Aspirin (100 mg) at least 4 days prior to the index intervention, alternatively at least 500 mg loading dose prior to or within 2 hours post procedure; Clopidogrel 75 mg daily at least 4 days prior to the index intervention, alternatively at least 300 mg loading dose prior to or within 2 hours post procedure (or ticlopidine, if required); the use of bivalirudin (Angiox™) was allowed as an alternative to heparin.

^2^Following the PTA procedure, subjects were to be prescribed daily acetyl-salicylic acid (ASA) (100 mg) indefinitely and daily clopidogrel (75 mg) (or ticlopidine, if required) for at least 1 month following the procedure. Prolonged antiplatelet therapy could be given at the discretion of the physician and should be considered after placement of stents.

^3^Within 6 weeks of procedure.

^4^For Angio Sub-group subjects only.

^5^If subject undergoes an unscheduled angiogram; a copy of the angiogram must be forwarded to the Angiographic Core Lab for adjudication.

^6^Only death and amputations.

^7^Wound assessment performed via the SilhouetteMobile.

^8^Only Wound Care.

### Data collection

Data will be collected via e-CRF during treatment at all investigational centers and will be completed prospectively during the hospital admission and follow-up.

### Study device

IA-DEB manufactured by Medtronic (Santa Rosa, CA, USA) features a 0.014” guidewire-compatible, over-the-wire drug-eluting balloon that uses paclitaxel as the therapeutic agent and urea as excipient. The characteristics and mode of action of the IN.PACT drug-eluting balloon have been described elsewhere [17,34,35].

## Discussion

While the body of clinical experience involving the treatment of CLI continues to evolve rapidly, our knowledge and understanding of the many factors that impact treatment outcomes of this heterogeneous and complex disease state remain incomplete. Nevertheless, there have been important recent contributions to our appreciation of factors affecting clinical outcomes, including the concept of angiosome-directed revascularization and recognition of the importance of restoring patency of the pedal run-off vessels. New technology has also provided a potential improvement over current modalities of therapy. A substantial treatment effect of the IA-DEB technology in improving infrapopliteal vessel patency and reducing re-intervention rates when compared to standard PTA has been reported at 3-, 6-, and 12-month time points by single-center registries [10] and randomized trials [18,19].

The reduction in re-intervention rates in this challenging CLI population is an undisputable hallmark of successful IA-DEB use; however, the important “patient-centric” outcomes of limb salvage, pain relief, mobility restoration, and improvement in quality of life remain equally important goals and endpoints in both clinical practice and research. Unfortunately, these fundamental “patient-centric” endpoints and essential post-treatment care and follow-up are infrequently pre-specified and/or are incompletely monitored and reported in CLI trials. Nevertheless, the three IA-DEB studies cited above reinforce the nature of the challenges in establishing a clear correlation between improved vessel patency and “patient-centric” outcomes. They also emphasize that successful CLI care goes beyond promoting durable vessel patency and is but one essential aspect of a multidisciplinary team approach, an approach that is representative of the IN.PACT DEEP trial design.

Therefore, as our clinical experience in the multifaceted care and treatment of CLI patients evolves, the IN.PACT DEEP stands as a unique milestone robust trial, which will contribute to our fundamental understanding of the role of durable vessel patency and its influence on “patient-centric” outcomes targeting the full spectrum of Rutherford class 4-5-6 critical limb ischemia and the identification of clinical and anatomical profiles that will derive the optimal benefit from DEB therapy.

## Trial status

In.PACT DEEP completed subject enrollment/randomization in July 2012, and the final enrolled subjects returned for clinical follow-up in August 2013. Public presentation of the trial co-primary and secondary endpoints is expected in 2014.

## Conclusion

The IN.PACT DEEP represents the first completed, largest, prospective, multicenter, randomized, controlled comparison of plain old balloon angioplasty to drug-eluting balloon angioplasty in infrapopliteal arteries of patients with CLI. The trial design is rigorous and provides unbiased assessment of outcomes through independent angiographic and wound-healing core labs and clinical event committee adjudication. IN.PACT DEEP tests the hypothesis that the IN.PACT Amphirion paclitaxel drug-eluting balloon provides superior 1-year vessel patency, assessed by late lumen loss (LLL), and whether any potential improvement in vessel patency is associated with a significant reduction in clinically driven target lesion revascularization through 1 year. Clinical follow-up is planned through 5 years and will continue to yield important information regarding the long-term impact of these two interventional strategies and set a new standard for clinical evidence that will assist physicians and caregivers with important direction in the care of this complex and challenging patient cohort.

## Abbreviations

ADE: Adverse device effect; AE: Adverse event; AFS: Amputation-free survival; Angio-TL: Angiographic target lesion; CEC: Clinical events committee; CLI: Critical limb ischemia; DEB: Drug-eluting balloon; DSMB: Data safety monitoring board; e-CRF: electronic clinical report form; IA-DEB: IN.PACT Amphirion drug-eluting balloon; ITT: Intention to treat; LLL: Late lumen loss; MAE: Major adverse event; PTA: Percutaneous transluminal angioplasty; QoL: Quality of life; SADE: Serious adverse device effect; SAE: Serious adverse event; TER: Target extremity revascularization; TL: Target lesion; TLR: Target lesion revascularization; UADE: Unanticipated adverse device effect; US IDE: United States investigational device exemption; WRA: Wound-related artery.

## Competing interests

AM and TZ are consultants for Medtronic; FM receives grant support from Medtronic. DS and ML are Medtronic employees. All other authors have nothing to disclose.

## Authors’ contributions

TZ, IB, DS, and ML conceived and participated in the design of the study. MBR, MBO, AM, PP, and FV contributed to the design of the study. ML drafted the manuscript. All authors approved the final manuscript.
